# PITHIA: Protein Interaction Site Prediction Using Multiple Sequence Alignments and Attention

**DOI:** 10.3390/ijms232112814

**Published:** 2022-10-24

**Authors:** SeyedMohsen Hosseini, Lucian Ilie

**Affiliations:** Department of Computer Science, The University of Western Ontario, London, ON N6A 5B7, Canada

**Keywords:** protein interaction site prediction, multiple sequence alignment, deep learning attention

## Abstract

Cellular functions are governed by proteins, and, while some proteins work independently, most work by interacting with other proteins. As a result it is crucially important to know the interaction sites that facilitate the interactions between the proteins. Since the experimental methods are costly and time consuming, it is essential to develop effective computational methods. We present PITHIA, a sequence-based deep learning model for protein interaction site prediction that exploits the combination of multiple sequence alignments and learning attention. We demonstrate that our new model clearly outperforms the state-of-the-art models on a wide range of metrics. In order to provide meaningful comparison, we update existing test datasets with new information regarding interaction site, as well as introduce an additional new testing dataset which resolves the shortcomings of the existing ones.

## 1. Introduction

Biological systems in a cell are controlled by proteins, and while many proteins function independently, the majority work in conjunction with others to function properly. The term protein-protein interaction (PPI) refers to physical contact between two or more protein molecules arising from a combination of electrostatic forces, hydrogen bonds, and hydrophobic interactions. The residues of the sequence of proteins that facilitate the connection between them are referred to as interaction sites.

Detecting the interaction sites of proteins will help researchers understand the mechanism of various biological processes, disease development, and drug design [1]. Protein-protein interactions play a preponderant role on the functionality of proteins, and it is because of this that some databases like PDB [2] or Uniprot [3] include information about the interactions and interaction sites. Other databases, such as PiSite [4], contain exclusively protein interaction sites.

Interaction site prediction can be achieved experimentally [5,6] or computationally [7,8]. Experimental methods are expensive, time consuming, and label intensive. Therefore, computational methods are prevalent. Computational models can be further classified into sequence-based methods [1,9,10], that rely only on the protein sequences, and those using also the protein structure as input [11,12,13]. Considering the fact that the number of existing protein sequences outnumbers the number of available structures by two orders of magnitude [14], sequence-based methods have the advantage of being much more widely applicable. For this reason, our new method, PITHIA, belongs to this category.

In recent years, machine learning methods and deep learning models have been extensively employed and have shown a great promise for applications in the field of protein interaction site prediction. Specifically, in sequence-based models, features like position-specific scoring matrix (PSSM), evolutionary conservation (ECO), putative relative solvent accessibility (RSA) have been used extensively in numerous models. With respect to machine learning models, support vector machine (SVM) classifiers [15,16], random forests [17] or the combination of the two [18] have been studied and have achieved good results. There exist multiple models that use recurrent neural networks (RNN) to improve the predictions, such as DLPred [1] that uses SLSTM, a modified version of the long short-term memory architecture (LSTM). Although convolutional neural network (CNN) models are mostly used in the field of image recognition, a number of studies have demonstrated that it could be effective for prediction of interaction sites. In addition to using structural information as input, a hybrid method like DeepPPISP [13] that combines a CNN and a multilayer perceptron (MLP), can further improve the performance of the model. Furthermore, this architecture processes entire sequences to enhance prediction. DELPHI [9] combines both CNN and RNN to further improve the performance. Additionally, it also adds some new features to the model like HSP [19], position information, and a reduced 3-mer amino acid embedding, ProtVec1D [20] and exhibits their effectiveness. Finally, SCRIBER [10] uses only logistic regression, yet provides a successful model due to its cross-prediction minimization approach.

Representing words as vectors in natural language processing has shown that their meaning can be captured in vector embeddings, such as those produced by word2vec [21] or GloVe [22]. Such embeddings can represent meaning in a very convenient way, since operations on vectors have a direct relationship with meaning; for example, vector(king)−vector(man)+vector(woman) results in a vector closest to vector(queen) [21]. Fixed embeddings were soon replaced by the more effective contextual ones, such as BERT [23] and ELMo [24]. BERT uses the very efficient attention model of transformers [25]. In recent years, there have been multiple models that tried to represent amino acids with embedding vectors employing the similar algorithms that exist in NLP [26,27,28].

Sequence alignment is the most widely used procedure for extracting useful information from sequences. Multiple sequence alignment (MSA) can identify deeply hidden relations between genes and evolutionary patterns by detecting structural or functional similarities among proteins in the same family. In particular, it could be used for identifying interaction sites. The powerful MSA has been combined with the highly successful attention learning model of transformers [25] to produce the MSA-transformer [26].

We introduce in this paper our new model, PITHIA, that uses the embeddings computed by the MSA-transformer in combination with an attention-based deep learning architecture to produce the most efficient model to date. PITHIA surpasses the current state-of-the-art programs by a wide margin. We prove this by throughly testing and comparing the programs on several of the most widely used datasets. Along the way, we update the older datasets with more recent and complete interaction site information, as well as design a new dataset that is the largest and most suitable for testing.

## 2. Results

### 2.1. Competing Methods

As our method relies only on sequence information to predict interaction sites, we compare it with the state-of-the-art sequence-based methods; meaning that they do not use any structural information to make their predictions. As the competing methods needed to be run on the new datasets, only methods with working web-server or available code were considered. As a result we have made comparisons with the following state-of-the-art methods: DLPred [1], SCRIBER [10], and DELPHI [9].

Two methods based on extreme gradient boosting [29,30], were not included because we could not test their performance; their code is not available and the authors have not responded to our inquiries.

### 2.2. Evaluation Metrics

As a good practice for evaluating binary classification performance, and as used in previous studies, we employ many metrics: sensitivity (or recall), precision, specificity, accuracy, F1-score, Matthew’s correlation coefficient (MCC), area under the receiver operating characteristic curve (ROC) and area under the precision-recall curve (PR).

Denoting by TP and TN the number of correctly predicted interaction sites and non-interaction sites, respectively, and by FP and FN the number of the incorrectly predicted interaction sites and non-interaction sites, respectively, sensitivity (recall), precision, specificity, accuracy, F1-score, and MCC are defined as follows:

$$Sens=\frac{TP}{TP+FN},\;\;Prec=\frac{TP}{TP+FP},\;\;Spec=\frac{TN}{TN+FP}$$

$$Acc=\frac{TP+TN}{TP+TN+FP+FN},\;\;F1=2\times \frac{Sens\times Prec}{Sens+Prec}$$

$$MCC=\frac{TP\times TN-FP\times FN}{\sqrt{\left(TP+FP)(TP+FN)(TN+FP)(TN+FN\right)}}$$

All metrics above, except the areas under ROC and PR curves, depend on the threshold used to separate positive and negative predictions. This threshold was chosen such that the number of predicted interaction residues equals that of actual interaction residues. This means TP+FP=TP+FN, which implies that FP=FN, which makes sensitivity equal to precision. Additionally, since the F1-score is the harmonic mean of sensitivity and precision, when the two are equal, this is also the values of the F1-score. In all tables below, for visual convenience, we still provide separate columns for sensitivity, precision, and F1-score, however, all these values are the same for each test.

As seen in Section 3.1, our datasets are highly imbalanced. Of the two curves, ROC and PR, it is the PR curve which better represents the performance of various methods for skewed data [31]. Note also that both curves do not depend on any threshold for their value, thus giving a better overall view of the method’s performance. The connection between the PR curve and our threshold described above is that a good PR curve is far away from the origin and the threshold represents the intersection point between the PR curve and the main diagonal. It tells therefore how far from the origin the curve is along the main diagonal.

### 2.3. Performance Comparison

We are now ready to compare our PHITIA model with SCRIBER, DLPred, and DELPHI. We use all test datasets in Section 3.1. As mentioned by [9], the training set of DLPred has significant similarities with Dset_448, thus preventing the inclusion of DLPred on this dataset. To enable comparison on this dataset, a subset was created by [9], Dset_335, containing the 355 proteins of Dset_448 that have no significant similarity with the training set of DLPred. We include comparison on this dataset as well.

The results are presented in Table 1. PITHIA outperforms the competition in all tests. Two of the most relevant parameters are the area under the PR curve and Matthew’s Correlation Coefficient (MCC). The advantage over the second best program with respect to the area under the PR curve is as high as 34.9%, with an average of 16.9%, whereas the improvement over the second best with respect to MCC goes up to 62.6%, with an average of 29.4%. For our most important dataset, Dset_500, the improvements over the second best are close to maximum: 29.2% for area under PR curve and 56.8% for MCC.

**Figure 1:**
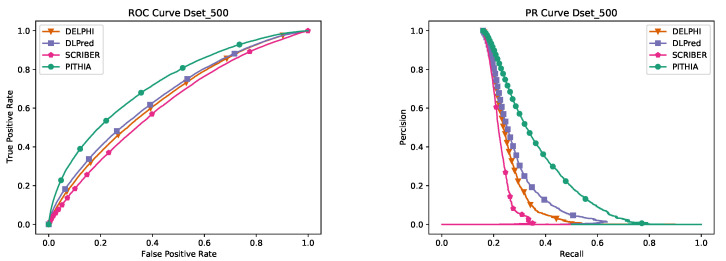

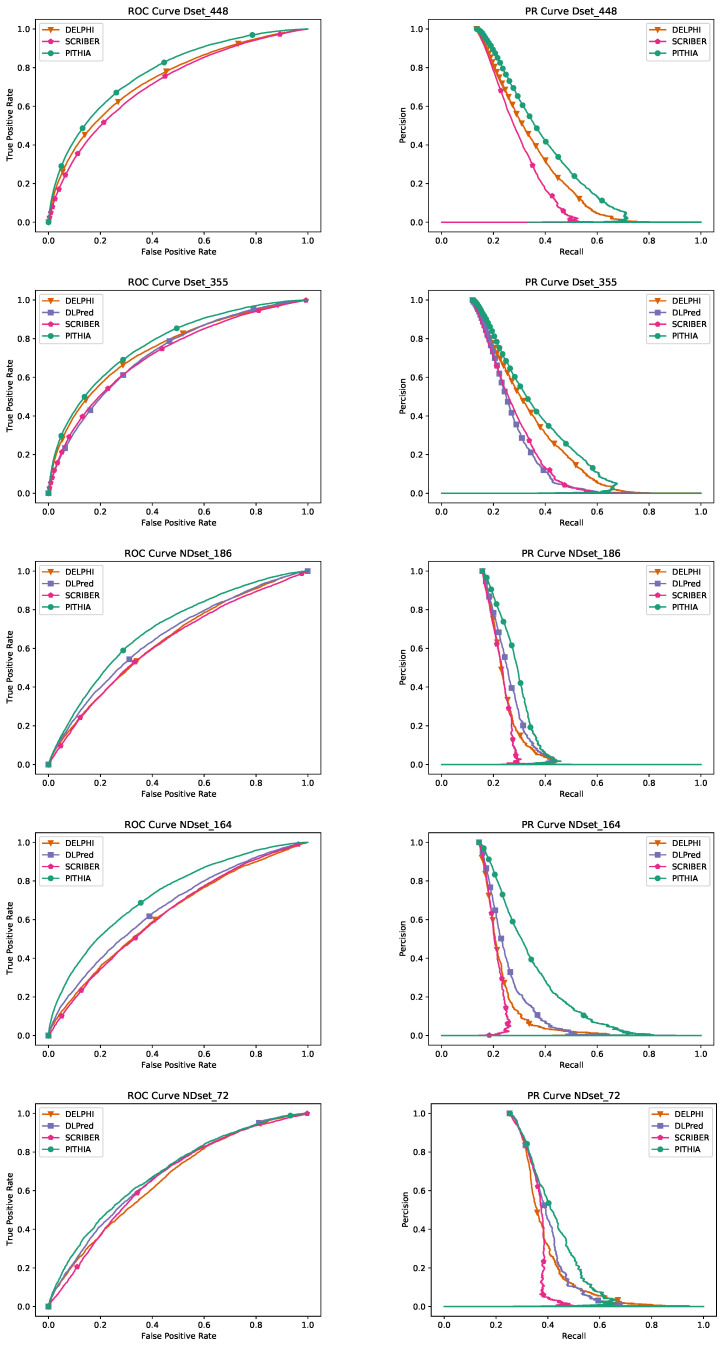
ROC and PR curves for the tests in Table 1, in the same order. The left column contains and ROC curves and the right one the PR curves. The two curves for each test are in the same row.

We present as well the ROC and PR curves for all datasets in Figure 1. The curves of PHITIA are always the highest, in some cases much higher than the rest.

It is also of interest to investigate the performance of the programs with respect to the protein lengths. The majority of proteins have less than 400 amino acids in their sequences. As a consequence, most of existing algorithms try to increase their performance on proteins with less than 400 amino acids, and some algorithms either completely ignore proteins that are too long (e.g., DeepPPISP [13] only supports proteins that have less than 500 amino acids) or do not perform well on those proteins. We combined all of the testing datasets, Dset_500, Dset_355, NDset_186, NDset_164, and NDset_72, into a single dataset and divided them according to the sequence length into five bins: 0–200, 200–400, 400–600, 600–800, and more than 800.

Table 2 gives the results for the top three programs. The first thing to notice is that PITHIA performs the best in all length intervals for all metrics. Second, the performance of all models decreases with protein length, confirming that longer proteins are harder to predict correctly. PITHIA outperforms the competition in the most difficult category of the long proteins by a very large margin: the area under PR curve is larger than that of DELPHI and DLPred by 21.4% and 43.4%, resp., whereas the MCC is better by 105% and 230%, resp.

## 3. Materials and Methods

### 3.1. Datasets

The majority of datasets which have been used for either training or testing are outdated due to numerous changes in the protein sequences and interaction sites. Consequently, it is critical to use updated information if we want to create a more accurate model. To update the older datasets and to create a new one, we used the most recent version (January 2019) of protein interaction residue chains provided by the PiSite database [4].

The existing datasets we consider are Dset_72 [32], Dset_164, Dset_186, [33], and Dset_448 [10]. Dset_448 is fairly recent but the others are not. We updated all datasets except Dset_448 with the most recent information in PiSite, to obtain the new versions: NDset_72, NDset_164, NDset_186. The first step was to search the PDB database for each sequence of the old datasets and match it with the provided ID. In case of multiple matches, we chose the ID of the sequence that has the highest similarity with the given sequence. After matching the IDs with their corresponding sequences, we searched PiSITE for the IDs and we updated the sequences and the interaction sites accordingly.

We also created a completely new testing dataset, Dset_500, as follows. We took all 22,654 proteins from PiSite and removed all sequences with no interaction residues or containing less than 50 amino acids; 14,203 sequences remained after this elimination. We then used PSI-CD-HIT [34,35] to detect and remove all sequences that have at least 25% similarity with any sequence in the training datasets of the main competing programs. After this we retain only the 2985 sequences that are alone in their clusters, from which we randomly selected 500 proteins to form Dset_500. Note that the sequences in Dset_500 not only have no similarities with any sequences in the other datasets but also with each other. This makes Dset_500 not only the largest but also the most dissimilar dataset, which makes it the most suitable for testing.

The process of creating a training dataset is described next. Starting with the 14,203 protein sequences obtained above, we use PSI-CD-HIT to remove any sequences that have any similarity above 25% with any of the testing datasets, NDset_72, NDset_164, NDset_186, Dset_448, Dset_500. The remaining proteins, 11,523, form PITHIA’s training dataset; this is further split into training (80%) and validation (20%) sets.

Table 3 gives an overview of all datasets.

### 3.2. Input Features

#### 3.2.1. Embeddings

Recently, a lot of attention has been given to protein language models that can capture various protein properties from unsupervised learning on millions of sequences [26,36,37]. The embeddings computed using transformer architectures outperform older methods in many applications. Transformers attention was coupled with the most widely used tool in bioinformatics, multiple sequence alignment, to produce the best protein folding prediction model, AlphaFold [38]. However, this was completed in a supervised manner. The first unsupervised use of multiple sequence alignment with attention is in the MSA-transformer of [39], which creates a 768-dimensional vector for each amino acids. These embeddings are the main feature used by our model.

The multiple sequence alignments are computed using HHblits [40] on the UniRef-50 database [41] dated 2020-03. Default settings are used except for HHblits’s number of search iterations (-n), which is instead set to 4. With this change, the algorithm will be able to find more sequences which could be useful for those proteins that might not have enough sequences. At the same time, since the result is ordered based on the similarity, increasing the number of iterations does not affect the quality of the sequences for the majority of the proteins.

The MSA-transformer, by default, uses the first 128 sequences to create the embedding. We have tested and compared several values for the number of sequences: 32, 64, and 128. For those proteins which total number of sequences in their MSA were less than the number required by the algorithm, the embeddings were computed with the sequences available, in order to enable testing of the model on any protein sequence.

#### 3.2.2. Other Features

The main feature of our model is the embeddings created by the MSA-transformer, but we study also the impact of traditionally used features, outlined below.

*PSSM:* Position-Specific Scoring Matrix (PSSM) represents protein sequences in an intuitive and highly informative way. As a result, PSSM-based feature descriptors have proven effective in improving the performance of a variety of protein attribute predictors. PSI-Blast [42] is used to compute the PSSM matrices with the number of iterations set to 3. Using PSI-Blast, each input sequence is aligned multiple times against the non-redundant database.

*Physicochemical characteristics:* These characteristics include three aspects of each amino acid: atom count, electrostatic charge and hydrogen bond potential [10].

*Evolutionary Conservation (ECO):* It reflects the fact that genes, regions of genes, or chromosome segments are conserved among species, not only demonstrating the common ancestry of species, but also implying a functional characteristic of the conserved element. The ECO score which is a one dimensional feature is computed with the same procedure that has been described in [10].

*RSA:* For determining protein folding and stability, the accessible surface area (ASA) of proteins has long been regarded as one of the main factors. ASA is commonly expressed in terms of RSA (Relative Solvent Accessibility). This is a one dimensional feature which is predicted using ASAquick [43].

*RAA:* An AA interaction propensity is defined by the relative abundance of a given AA type in comparison to the corresponding noninteraction residues on the protein surface. This is a one dimensional feature which is computed with the same formula presented in [44].

*Hydropathy index:* An amino acid’s hydropathy index is a number that indicates whether its sidechain is hydrophobic or hydrophilic [45]. Generally, the larger the number, the more hydrophobic the amino acid.

*PK_x_:* Dissociation constants measure the propensity of a molecule to dissociate into its constituent parts [46]. There are three different dissociation constants: PK_a_ is the negative of the logarithm of the dissociation constant for the -COOH group, PK_b_ is the negative of the logarithm of the dissociation constant for the -NH3 group, and PK_x_ is the negative of the logarithm of the dissociation constant for any other group in the molecule. This paper follows the same patterns that other papers follow and it only considers PK_x_ as a side feature.

*Putative protein-interaction disorder:* Functional elements of disordered proteins play a significant role in protein-protein interactions. This is a one dimensional feature which is computed using the ANCHOR program [47].

*Physical properties:* Each amino acid type is assigned a 7D property. Among them are graph shape index, polarizability, volume (normalized van der Waals volume), hydrophobicity, isoelectric point, helix probability, and sheet probability. Pre-computed values are taken from [10].

To improve the convergence of the model, the values of the feature vectors are normalized, after they have been computed, using the min-max normalization formula:

$$v_{\mathrm{norm}}=\frac{v-v_{\min}}{v_{\max}-v_{\min}}\;.$$

Many papers have evaluated the aforementioned features, and all of them found that these features were able to increase prediction accuracy. ECO, PSSM, and multiple sequence alignment, on the other hand, use HHbilts as their core layer. Consequently, it is expected that the embeddings created by the MSA-transformer already incorporate ECO and PSSM information to some extent. Furthermore, ref. [37] shows that the embeddings are capable of understanding the physicochemical and physical characteristic of the protein residues. It is therefore interesting to investigate how much information these features can add to the MSA-transformer embeddings.

### 3.3. Model Architecture

**Figure 2:**
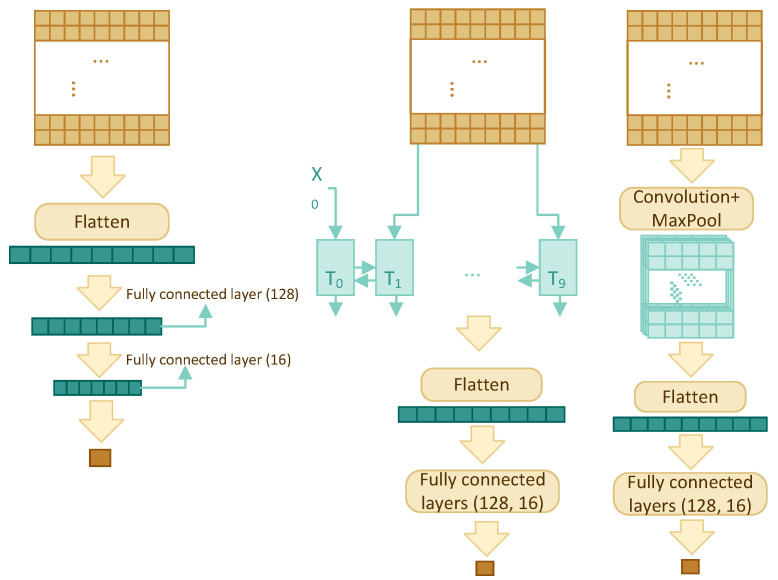
The MLP, RNN, CNN architectures (from left to right).

**Figure 3:**
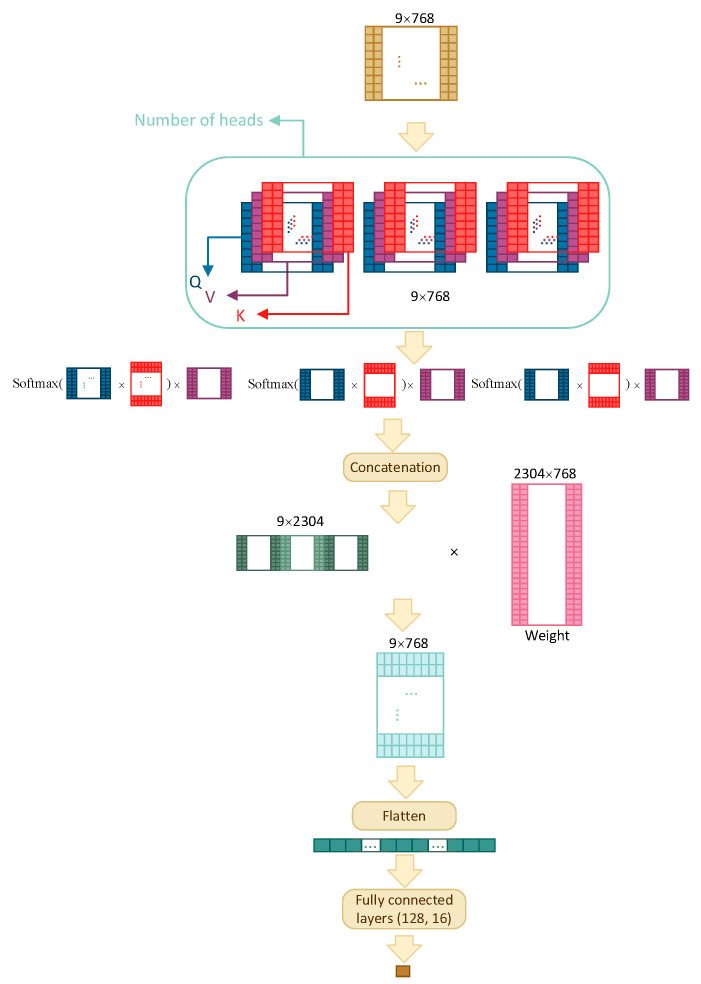
The TF architecture with three attention heads.

Four different architectures have been compared in order to find the best model: multilayer perceptron (MLP), recurrent neural network (RNN), convolutional neural network (CNN), and transformer self-attention (TF). The architectures are shown schematically in Figure 2, for the first three architectures, and Figure 3, for TF, and discussed in detail below.

All architectures accept a 2-dimensional input of size w×768, where *w* is window size, and output a single value between 0 and 1, which is the predicted interaction likelihood of the residue residing in the centre of the window.

#### 3.3.1. Multilayer Perceptron

This is the simplest yet one of the most effective models in this paper. It uses the sliding window and the concept of many-to-one. The model consists of a flatten layer and three fully connected layers with dropout for regularization. The w×768 input is flattened and fed into a fully connected layer to reduce its size to 128, then everything passes through two more fully connected layers to reduce the dimension to one.

#### 3.3.2. RNN

The RNN component consists of a bidirectional GRU layer, a flatten layer, and two fully connected layers. A bidirectional GRU layer is applied to the w×768 feature profile with the intention of storing the dependency and relationship among the *w* residues. Instead of returning a single value, we have set the GRU layer to return an entire sequence. Flattened results are fed into two fully connected layers with dropout to reduce the dimension to one.

#### 3.3.3. CNN

The two-dimensional (2D) CNN model has one convolution layer, one max pooling layer, one flatten layer, and two fully connected layers with dropout. The sigmoid activation function is used in the last fully connected layer, so that the output is a single number between 0 and 1.

In contrast to two-dimensional CNN’s which are mostly used for images, one-dimensional (1D) CNN’s are used for text and one-dimensional signals. The architecture of one-dimensional CNN is similar to the 2D CNN; it has one convolution layer, one max pooling layer, one flatten layer, and two fully connected layers.

#### 3.3.4. Self-Attention

One of the important parts of Transformers is self-attention. The TF model consists of a multihead attention layer, a flatten layer, and three fully connected layers with dropout. If side features are used (other than embeddings), then the size of input is reduced to w×128 before concatenating the side features.

As illustrated in Figure 3, the input is of size w×768 and the attention layer, depending on the number of heads (three, in the figure), creates multiple sets of Query, Value, and Key matrices. These matrices are the results of multiplication between embeddings and different weight matrices whose values are tuned through training. The model then uses the following equation to capture information from each head:

$$Attention\left(Q,K,V\right)=Softmax\left(\frac{QK^{T}}{\sqrt{d_{k}}}\right)V\;.$$

The results from all heads are then concatenated and multiplied with a weight matrix that is trained jointly with the model; the final output is flattened and is fed to three fully connected layers with dropout before obtaining the final result.

### 3.4. Implementation

The program is written in Keras [48] (Python 3.6.2) with TensorFlow GPU [49] as a backend. In order to process the sliding windows for each amino acid in a sequence, since each amino acid is embedded into a vector of size 768, it would have required around 700 GB of space to be able to fit the whole dataset in the RAM. In order to mitigate this problem, we used generators to build up batches on the fly. Generators are special types of functions that return a sequence of values instead of an individual value. They allow the program to load only the amount of data necessary for the current epoch, instead of loading the entire dataset. Although this method decreases the required space in memory, it will increase the I/O and it will not let the program use the architecture to its full potential. One of the best practices in deep learning is to shuffle the whole dataset before feeding it to the architecture. For our specific problem, this is crucial due to the nature of the data. When each window is extracted using the sliding window, adjacent windows are very similar—only the first and last residues are different. Shuffling the entire training dataset makes each batch more heterogeneous. This shuffling might create a batch that each of its instances comes from a different protein. Since the input file expected to be a FASTA format and with the generators the program only reads small parts of the input file, we could not use global shuffling, instead, we used local shuffling. We have increased the batch size to 1024 so that, considering that most proteins have fewer than 400 amino acids in their sequence, using a batch size of 1024 makes it possible to have multiple proteins in each batch.

In the end, we have managed to both reduce the memory and properly prepare the data for learning. With this method, it was possible to generate the dataset on multiple cores in real time and feed it into the deep learning model. Our architecture is able to be run on a server with 64 Gigabytes of RAM, 12 CPU cores, and one GPU (model T4 with 16 Gigabytes of VRAM). It takes about 24 h to train the model for 100 epochs. Testing takes about 10 s per sequence if embeddings are available, and about 10–20 min to compute the embeddings.

### 3.5. Selecting the PITHIA Model

A number of models have been built and compared using the training dataset; each model was trained on 80% of the data and then its performance was measured on the 20% validation data. The model with the best performance, measured as the area under the PR curve, on the validation data was chosen as the final PITHIA model. (No testing data was used in training any of the models discussed.)

We considered the four architectures mentioned above, with various parameter combinations from Table 4, as well as several multiple alignment sizes. The final PITHIA model was the transformer self-attention model with one head, using a sliding window of size w=9 over a sequence with zero padding at the ends, whose 9×768 input consists of the 768-dimensional MSA-Transformer embeddings corresponding to the 9 residues in the sliding window.

#### 3.5.1. Model Robustness

After establishing our model using the validation data, we check its robustness by comparing a number of models on the Dset_500 dataset. The results are shown in Table 5 where various architectures, window sizes, number of heads, and multiple alignment sizes have been considered. The final PITHIA model outperforms all the other candidates. It is interesting to remark that while the original paper [26] suggests using 128 sequences would create the best predictions for their purpose, our best model was obtained using 64 sequences.

Interestingly, the second best model in Table 5 is the MLP with the same window size as PITHIA. We have performed another test comparing only these two models on a different dataset, Dset_448. The results in Table 6 show that our PITHIA model outperforms more clearly the MLP contender.

#### 3.5.2. Side Features

We have tested the impact of adding some or all of the other features, in addition to the MSA-transformer embeddings. Some of the results are shown in Table 7. It is clear from the results, particularly in the PR column, that there is practically no improvement due to adding these features. Therefore, for simplicity, as well as avoiding the need to compute these features, we have decided to keep as input features the 768-dimensional embeddings alone. We note that, in agreement with [39], the embeddings appear to already contain the information provided by all the other features investigated.

Due to the large difference between the amino acid embedding size of 768 and the small dimension of a side feature—e.g., PSSM has dimension 20—a fully connected layer is added to the model after the self-attention layer in order to reduce the 768 dimension to 128 before concatenation with the side features.

## 4. Discussion

In this paper we propose a novel approach to the problem of protein interaction site prediction using embeddings produced by the MSA-transformer and a self-attention-based architecture. Our new model, PITHIA, clearly outperforms the state-of-the-art methods. We show this by extensive testing on multiple datasets, updated and a newly created one, largest and most relevant for testing.

In view of the above results, the old wisdom that says: “one or two homologous sequences whisper […] a full multiple alignment shouts out loud” [50], receives confirmation into yet another dimension: a choir of MSAs sings much louder than any single alignment.

Protein interaction site prediction is a fundamental problem and there is still a lot of room for improvement. Our new model makes use of very powerful concepts, multiple sequence alignments and attention, which make it work much better than the existing methods. The ideas used here should be useful for improving the prediction of proteins with other molecules, such as DNA, RNA, and other ligands.

### Availability

The trained model, source code and datasets are freely available for download at github.com/lucian-ilie/PITHIA (accessed on 21 September 2022).

The web server is available at pithia.csd.uwo.ca (accessed on 21 September 2022). It has been developed using Python Flask 2.1, Celery 5.2, and Redis 7.0 on Ubuntu 18.04 operating system. The user inputs protein sequences and receive the prediction results via e-mail. The average computation time for a protein of length 500 on the web server is 15 min.

## Figures and Tables

**Table 1:** Performance comparison with the state-of-the-art models SCRIBER, DLPred and DELPHI on datasets Dset_500, Dset_448, Dset_355, NDset_186, NDset_164, and NDset_72. The best results are shown in boldface.

Model	Sens	Prec	Spec	Acc	F1	MCC	ROC	PR
**Dset_500**								
DELPHI	0.281	0.281	0.864	0.772	0.281	0.145	0.649	0.256
DLPred	0.301	0.301	0.868	0.778	0.301	0.169	0.664	0.281
SCRIBER	0.248	0.248	0.858	0.761	0.248	0.106	0.620	0.224
PITHIA	**0.381**	**0.381**	**0.883**	**0.804**	**0.381**	**0.265**	**0.726**	**0.363**
**Dset_448**								
DELPHI	0.371	0.371	0.901	0.829	0.371	0.272	0.737	0.337
SCRIBER	0.334	0.334	0.896	0.821	0.333	0.230	0.715	0.287
PITHIA	**0.408**	**0.408**	**0.907**	**0.840**	**0.408**	**0.315**	**0.778**	**0.387**
**Dset_335**								
DELPHI	0.364	0.364	0.914	0.848	0.364	0.278	0.746	0.326
DLPred	0.308	0.308	0.906	0.835	0.308	0.214	0.724	0.272
SCRIBER	0.322	0.322	0.908	0.838	0.322	0.230	0.719	0.275
PITHIA	**0.381**	**0.381**	**0.916**	**0.852**	**0.381**	**0.297**	**0.762**	**0.344**
**NDset_186**								
DELPHI	0.267	0.267	0.863	0.770	0.267	0.131	0.639	0.243
DLPred	0.292	0.292	0.868	0.778	0.292	0.161	0.659	0.260
SCRIBER	0.262	0.262	0.862	0.768	0.262	0.125	0.629	0.229
PITHIA	**0.320**	**0.320**	**0.873**	**0.786**	**0.320**	**0.193**	**0.701**	**0.287**
**NDset_164**								
DELPHI	0.249	0.249	0.873	0.783	0.249	0.122	0.629	0.226
DLPred	0.277	0.277	0.878	0.791	0.277	0.155	0.660	0.252
SCRIBER	0.237	0.237	0.871	0.780	0.237	0.109	0.628	0.206
PITHIA	**0.360**	**0.360**	**0.892**	**0.815**	**0.360**	**0.252**	**0.731**	**0.340**
**NDset_72**								
DELPHI	0.384	0.384	0.793	0.690	0.384	0.176	0.658	0.387
DLPred	0.413	0.413	0.803	0.704	0.413	0.215	0.678	0.398
SCRIBER	0.382	0.382	0.792	0.689	0.382	0.174	0.662	0.359
PITHIA	**0.436**	**0.436**	**0.810**	**0.716**	**0.436**	**0.246**	**0.693**	**0.423**

**Table 2:** Length comparison on all datasets combined.

Lengths	Sens	Prec	Spec	Acc	F1	MCC	ROC	PR
**DELPHI**								
0–200	0.382	0.382	0.793	0.690	0.382	0.175	0.649	0.373
200–400	0.262	0.262	0.882	0.796	0.262	0.143	0.652	0.233
400–600	0.180	0.180	0.907	0.833	0.180	0.087	0.624	0.158
600–800	0.143	0.143	0.903	0.825	0.143	0.045	0.608	0.131
800–	0.137	0.137	0.920	0.854	0.137	0.057	0.617	0.117
**DLPred**								
0–200	0.387	0.387	0.795	0.692	0.387	0.182	0.650	0.384
200–400	0.296	0.296	0.887	0.805	0.296	0.183	0.687	0.261
400–600	0.225	0.225	0.912	0.842	0.225	0.137	0.663	0.186
600–800	0.148	0.148	0.903	0.826	0.148	0.051	0.604	0.132
800–	0.108	0.108	0.918	0.849	0.108	0.025	0.556	0.099
**PITHIA**								
0–200	0.450	0.450	0.815	0.724	0.450	0.265	0.710	0.449
200–400	0.343	0.343	0.895	0.818	0.343	0.238	0.725	0.319
400–600	0.272	0.272	0.917	0.852	0.272	0.189	0.709	0.238
600–800	0.255	0.255	0.915	0.848	0.255	0.171	0.694	0.223
800–	0.192	0.192	0.925	0.863	0.192	0.117	0.647	0.142

**Table 3:** Datasets parameters.

Dataset	Proteins	Mean	Total	Interaction	Interaction
	Sequences	Length	Residues	Residues	% of Total
Dset_500	500	274.54	137,270	21,222	15.5
Dset_448	448	260.05	116,500	15,810	13.6
NDset_186	186	209.72	39,008	6128	15.7
NDset_164	164	223.10	36,589	5276	14.4
NDset_72	72	211.46	15,236	3832	25.2
Training + Validation	11,523	251.90	2,902,667	545,724	18.8

**Table 4:** Parameter and hyper-parameters used for testing the algorithms.

Parameters	Values	
MLP		
Layer sizes	128, 16, 1	
CNN	1D	2D
Kernel size	3	5
Stride	1	1
Padding	same	valid
Number of filters	64	48
Window size	27, 35	25, 33
RNN		
GRU units	64	
Window size	35, 51, 63	
TF		
Number of heads	1, 2, 4	
Key size	2, 5, 9, 12, 31	
Window size	9, 17, 61	
All architectures		
Epochs	100	
Dropout	0.3	
Batch size	1024	
Optimizer	Adam $\left(\beta_{1}=0.9;\beta_{2}=0.999\right)$	
Loss function	Binary cross entropy	
Learning rate	0.001	

**Table 5:** Model comparison: W*w*, H*h*, and A*a* mean window size *w*, *h* attention heads, and multiple alignment size *a*, respectively. The best results are shown in boldface. * W9H1A64 is the final PITHIA model.

Model	Sens	Prec	Spec	Acc	F1	MCC	ROC	PR
**MLP**								
W5	0.375	0.375	0.882	0.802	0.375	0.257	0.722	0.353
W9	0.381	0.381	0.883	0.803	0.381	0.264	0.721	0.358
W17	0.372	0.372	0.882	0.801	0.372	0.254	0.714	0.347
W31	0.374	0.374	0.882	0.801	0.374	0.256	0.718	0.350
**RNN**								
W17	0.324	0.324	0.872	0.785	0.324	0.196	0.675	0.300
W25	0.327	0.327	0.873	0.786	0.327	0.200	0.679	0.305
W31	0.333	0.333	0.874	0.788	0.333	0.207	0.687	0.314
W39	0.328	0.328	0.873	0.787	0.328	0.201	0.679	0.292
**CNN-1D**								
W13	0.319	0.319	0.872	0.784	0.319	0.190	0.662	0.282
W19	0.323	0.323	0.872	0.785	0.323	0.196	0.658	0.289
**CNN-2D**								
W17	0.333	0.333	0.874	0.788	0.333	0.208	0.689	0.305
W19	0.342	0.342	0.876	0.791	0.342	0.218	0.698	0.316
**TF**								
W9H1A64 *	**0.381**	**0.381**	**0.883**	**0.804**	**0.381**	**0.265**	**0.726**	**0.363**
W9H2A64	0.374	0.374	0.882	0.801	0.374	0.256	0.721	0.353
W9H4A64	0.362	0.362	0.880	0.798	0.362	0.242	0.711	0.344
W9H1A32	0.367	0.367	0.881	0.799	0.367	0.248	0.707	0.351
W9H1A128	0.364	0.364	0.880	0.798	0.364	0.244	0.708	0.342
W5H1A64	0.364	0.364	0.880	0.798	0.364	0.245	0.718	0.346
W17H1A64	0.368	0.368	0.881	0.800	0.368	0.249	0.719	0.354
W31H1A64	0.329	0.329	0.874	0.787	0.329	0.203	0.682	0.348

**Table 6:** Comparison of two models, PITHIA (W9H1A64) and MLP-W9, on dataset Dset_448.

Model	Sens	Prec	Spec	Acc	F1	MCC	ROC	PR
PITHIA	0.408	0.907	0.408	0.840	0.408	0.315	0.778	0.387
MLP-W9	0.401	0.906	0.401	0.838	0.401	0.307	0.770	0.378

**Table 7:** Comparison the addition of side features. * The “None” row represents the PITHIA model.

Add Feature	Sens	Prec	Spec	Acc	F1	MCC	ROC	PR
PH	0.382	0.382	0.883	0.804	0.382	0.265	0.732	0.364
PSSM	0.383	0.383	0.884	0.804	0.383	0.267	0.729	0.366
ECO	0.386	0.386	0.884	0.805	0.386	0.270	0.730	0.367
PSSM + PH	0.382	0.382	0.883	0.804	0.382	0.265	0.726	0.365
PSSM + ECO	0.381	0.381	0.883	0.804	0.381	0.265	0.731	0.365
Anchor	0.381	0.381	0.883	0.804	0.381	0.264	0.728	0.363
HYD	0.380	0.380	0.883	0.803	0.380	0.263	0.726	0.365
RAA	0.374	0.374	0.882	0.801	0.374	0.256	0.724	0.359
All	0.379	0.379	0.883	0.803	0.379	0.262	0.729	0.365
None *	0.381	0.381	0.883	0.804	0.381	0.265	0.726	0.363

## Data Availability

All datasets are freely available at github.com/lucian-ilie/PITHIA (accessed on 21 September 2022).
